# Associations of *Helicobacter pylori* and hepatitis A seropositivity with asthma in the Hispanic Community Health Study/Study of Latinos (HCHS/SOL): addressing the hygiene hypothesis

**DOI:** 10.1186/s13223-021-00625-3

**Published:** 2021-11-24

**Authors:** Christian S. Alvarez, M. Larissa Avilés-Santa, Neal D. Freedman, Krista M. Perreira, Olga Garcia-Bedoya, Robert C. Kaplan, Martha L. Daviglus, Barry I. Graubard, Gregory A. Talavera, Bharat Thyagarajan, M. Constanza Camargo

**Affiliations:** 1 Division of Cancer Epidemiology and Genetics, National Cancer Institute, Rockville, MD USA; 2 Division of Clinical and Health Services Research, National Institute on Minority Health and Health Disparities, Rockville, MD USA; 3 Department of Social Medicine, UNC School of Medicine, Chapel Hill, NC USA; 4 Institute for Minority Health Research, University of Illinois at Chicago, Chicago, IL USA; 5 Academic Internal Medicine and Geriatrics, Department of Medicine , University of Illinois at Chicago, Chicago, IL USA; 6 Department of Epidemiology and Population Health, Albert Einstein College of Medicine, Bronx, NY USA; 7 Public Health Sciences Division, Fred Hutchinson Cancer Research Center, Seattle, WA USA; 8 South Bay Latino Research Center, Department of Psychology, San Diego State University, San Diego, CA USA; 9 Department of Laboratory Medicine and Pathology, University of Minnesota, Minneapolis, MN USA

**Keywords:** Asthma, *Helicobacter pylori*, Hepatitis A virus, Mexicans, Puerto Ricans

## Abstract

**Background:**

The hygiene hypothesis posits that microbial exposure reduces risk of asthma and other respiratory-related diseases. *Helicobacter pylori* and hepatitis A virus (HAV) are common fecal–oral infections. Our study aimed to examine associations of seropositivity to these agents with asthma in the Hispanic Community Health Study/Study of Latinos (HCHS/SOL).

**Methods:**

A total of 12,471 HCHS/SOL participants with baseline data on self-reported physician-diagnosed asthma, and antibodies anti-*H. pylori* and anti-HAV were included in this cross-sectional analysis. Multivariable logistic regression models were used to estimate the odds ratios and 95% confidence intervals for the overall associations of seropositivity to each agent with asthma. Analyses were also stratified by Hispanic/Latino background. Effect modification by smoking status and nativity were tested. An analysis restricted to individuals with spirometry-defined chronic obstructive pulmonary disease (COPD) was also considered.

**Results:**

The weighted overall prevalence of asthma was 16.6%. The weighted seroprevalence of *H. pylori* was 56.6% and of HAV was 76.6%, and they significantly differed by Hispanic/Latino background. After accounting for age, sex, education and other key confounders, we found no associations between *H. pylori* or HAV seropositivity with asthma (with and without COPD), either for all individuals combined or for any of the six specific backgrounds. There were no significant interactions by smoking and nativity.

**Conclusion:**

Our findings did not provide support for the role of *H. pylori* or HAV, as evidence of the hygiene hypothesis in asthma among the large and diverse Hispanic/Latino populations of the HCHS/SOL.

*Trial registration* NCT02060344

## Introduction

In recent decades, the prevalence of asthma has increased worldwide [1], whereas the prevalences of *Helicobacter pylori* and hepatitis A virus (HAV) infections have decreased. The hygiene hypothesis proposes that increased exposure to microbes in early life may be protective against asthma and associated allergic diseases [2].

Several epidemiologic studies in European and Asian populations have reported an inverse association between past exposure to *H. pylori* infection and the occurrence of asthma [3]. However, this association is controversial. More limited evidence also suggests an inverse link between exposure to HAV infection and asthma [4].

To the best of our knowledge, a few studies have examined the hygiene hypothesis regarding asthma among Hispanics/Latinos. A small study among Hispanic (n  = 57) and Chinese (n  = 150) immigrants to New York reported that none of infections assessed, including *H. pylori* (serology results) and hepatitis (self-reported data), were protective against the presence of asthma [5]. Another study among children (n  = 905; including 50 with history of asthma treatment) in Michigan found no association between larger family size—which influences the spread of fecal–oral infections, and asthma [6]. These previous studies are limited by a small sample size (≤ 50 individuals with asthma) and lack of ethnic diversity. In the US, the prevalence of asthma varies by race/ethnicity, disproportionately affecting African Americans and Hispanics/Latinos. A previous analysis within the Hispanic Community Health Study/Study of Latinos (HCHS/SOL) shows that asthma is more prevalent among Puerto Ricans than among other background groups, and among Hispanics/Latinos born in the US or who immigrated as children compared to those who immigrated as adults [7]. Additionally, our recent study showed that the seroprevalence of *H. pylori* infection is high (57%) within the HCHS/SOL population and varies by self-reported Hispanic/Latino background, ranging from 47% in Puerto Rican to 72% in Central American backgrounds [8]. Given the high prevalence of both asthma and *H. pylori* infection in Hispanics/Latinos, HCHS/SOL is an important setting to assess the hygiene hypothesis by examining associations between seropositivity to *H. pylori* and HAV and asthma.

## Methods

### Study population

Detailed information on the HCHS/SOL has been published [9]. Briefly, this cohort is an ongoing community-based probability sample study designed to evaluate risk factors of chronic diseases in a diverse Hispanic/Latino population. The HCHS/SOL study recruited 16,415 individuals aged 18–74 years from randomly selected households in four U.S. urban communities during 2008–2011. Questionnaires at the baseline examination were used to obtain information on demographics, health/medical history, social/language acculturation, smoking and alcohol habits. Clinical assessments such as dental exams were also performed at baseline, and a fasting sample of blood was collected. The HCHS/SOL was approved by institutional review boards at each participating institution and written informed consent was obtained from all participants.

For this cross-sectional analysis, we included 12,471 individuals with complete baseline data on asthma, *H. pylori,* and HAV serology. This study was approved by the HCHS/SOL Committee of Ancillary studies (AS#2016.08) and was exempted from institutional review board evaluation by the National Institutes of Health Office of Human Subjects Research Protection.

### Asthma and chronic obstructive pulmonary disease (COPD) ascertainments

Self-reported, physician-diagnosed lifetime asthma was defined by a positive answer to the following questions: “Have you ever had asthma?” and “Was it diagnosed by a doctor or other healthcare professional?” Physician-diagnosed asthma was also examined without a COPD diagnosis as both conditions have similar symptoms, are treated with bronchodilators, and asthma is sometimes mistaken for COPD. COPD was defined by a postbronchodilator spirometry FEV1/FVC ratio  < 0.70 [10]. Spirometry was conducted in accordance with American Thoracic Society/European Respiratory Society guidelines [11].

As secondary analyses in individuals without COPD, we also examined the associations with current asthma (physician-diagnosed asthma with the use of an asthma treatment within the previous 12 months), childhood onset of asthma (physician-diagnosed asthma at age 15 or younger), wheeze (ever having had an attack of wheezing or whistling in your chest that made you feel short of breath in the last 12 months) and hay fever ever (ever having had hay fever).

### Laboratory assessments for *H. pylori* and HAV infections

Anti-*H. pylori* immunoglobulin G antibodies were measured in baseline plasma using a commercial whole-cell enzyme-linked immunosorbent assay kit (Biohit, Helsinki, Finland). As previously reported [8], seropositivity was defined as titers  >  30 EIU/ml.

Total anti-HAV antibodies were measured in baseline sera using direct chemiluminescent technology on the ADVIA Centaur System (Siemens Healthcare Diagnostics, Deerfield, Illinois, US). Seropositivity was defined as values  ≥ 1.

Both serological assessments were performed in the central HCHS/SOL laboratory at the University of Minnesota. Staff was blinded to any participant characteristics.

### Statistical analysis

The seroprevalences of asthma, *H. pylori*, and HAV were estimated for all participants and by Hispanic/Latino background. Descriptive statistics were computed for all asthmatic individuals and those without COPD. Logistic regression models were used to estimate the odds ratios (ORs) and 95% confidence intervals for the associations of each infectious agent with asthma. Using a complete case dataset, models were adjusted for Hispanic/Latino background, age, sex, education, nativity, language acculturation, cigarette smoking, number of missing teeth, number of doctor’s visits, ferritin levels, and body mass index. Most of these factors are significant determinants of *H. pylori* seropositivity in this cohort [8]. A more parsimonious model excluding number of missing teeth, number of doctor’s visits, ferritin levels was also considered. The covariables with the highest proportion of missing data were missing teeth (7%), ferritin (2%), doctor’s visits (1%), nativity (0.3%), and language acculturation (0.2%). Analyses were also stratified by Hispanic/Latino background.

For both *H. pylori*- and HAV-asthma associations, we assessed effect modification by smoking status (never, former light, current light, former intermediate, current intermediate, former heavy and current heavy smokers) and nativity [US born (50 states and DC), foreign born with  <  10 years in the US, foreign born with  ≥  10 years in the US]; and product terms were evaluated by a Wald test. All p values are two-sided and a p value  ≤ 0.05 was considered statistically significant without adjustment for multiple comparisons.

All analyses were performed using sample weights and the other aspects of the complex sample design via the survey analysis procedures in SAS 9.4 software (SAS Institute Inc., Cary, NC) and SUDAAN 11.0.3 software (Research Triangle Institute, Research Triangle Park, NC).

## Results

**Fig. 1:**
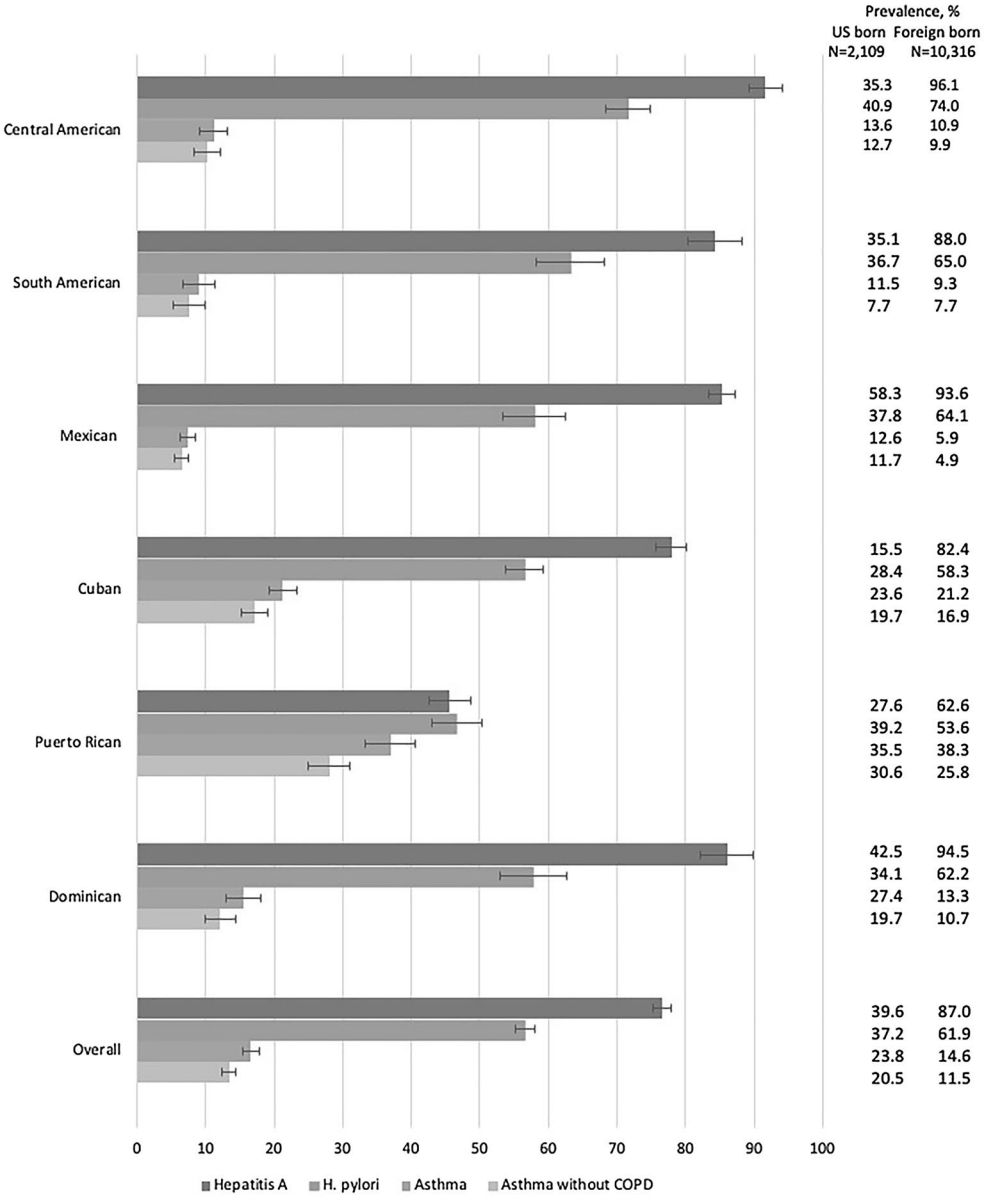
Overall and Hispanic/Latino background-specific weighted prevalences of Hepatitis A virus, *Helicobacter pylori*, asthma and asthma without COPD

The weighted prevalence of physician-diagnosed asthma was 16.6%, ranging from 7.4% among Mexicans to 36.9% in Puerto Rican background (Fig. 1). The weighted seroprevalence of *H. pylori* was 56.6% and of HAV was 76.6%. Seroprevalence to both agents differed by Hispanic/Latino background. Notably, Puerto Rican background had lower seroprevalences of both *H. pylori* (46.7%) and HAV (45.6%) (Fig. 1).

Table 1 shows selected baseline characteristics of all participants, asthmatics and asthmatics without COPD. Overall, the median age was 40 years, nearly one-third reported having less than high school education and  ~ 78% were foreign born. In addition, one-third reported not seeing a doctor in the last year, 40% were obese, and 21% were current smokers. The median ages were 40 [Interquartile range (IQR): 27, 52] and 36 (IQR: 25, 49) for individuals with asthma and for those with asthma without COPD, respectively. Among the individuals in the latter group, 59% were women, two-thirds were foreign born, and 29% reported having less than a high school education. In addition, 25% reported not seeing a doctor in the last year, 50% were obese and 25% were current smokers.

**Table 1:** Baseline participant characteristics overall and by asthma

	Total N = 12,471	Physician-diagnosed asthma^c^ N = 1937	Physician-diagnosed asthma without COPD^d^ N = 1548	Physician-diagnosed childhood onset of asthma^e^ N = 894
	No. (%^b^)	No. (%^b^)	No. (%^b^)	No. (%^b^)
Age, median (Q1, Q3)	40 (28, 52)	40 (27, 51)	36 (25, 49)	31 (23, 43)
Sex				
Female	7450 (52.1)	1313 (58.6)	1073 (58.7)	540 (50.4)
Male	5021 (47.9)	624 (41.4)	475 (41.3)	354 (49.6)
Background^a^				
Cuban	2037 (22.6)	428 (29.0)	326 (28.8)	249 (34.6)
Dominican	1173 (10.2)	187 (9.5)	146 (9.2)	80 (7.4)
Mexican	4841 (36.6)	358 (16.3)	303 (17.7)	135 (15.7)
Puerto Rican	1914 (14.5)	657 (32.2)	504 (30.1)	249 (25.9)
Central American	1302 (7.2)	150 (4.9)	135 (5.5)	90 (6.1)
South American	830 (5.0)	72 (2.8)	62 (2.9)	33 (2.5)
Others	359 (3.9)	85 (5.3)	72 (5.8)	58 (7.8)
Education level^a^				
Less than high school	4654 (31.9)	659 (29.6)	506 (28.6)	241 (24.8)
High school or equivalent	3216 (28.1)	520 (28.8)	427 (30.3)	268 (32.4)
Greater than high school or equivalent	4577 (40.0)	757 (41.6)	614 (41.1)	385 (42.8)
Nativity^a^				
US born (50 states + DC)	2109 (21.9)	540 (31.3)	446 (33.3)	305 (37.5)
Foreign born (< 10 years in US)	3016 (28.9)	397 (25.2)	317 (25.1)	226 (28.5)
Foreign born (≥ 10 years in US)	7300 (49.2)	995 (43.5)	781 (41.6)	359 (34.0)
Acculturation SASH-LANG score^a^				
Low (1–2)	9920 (74.5)	1266 (60.4)	1003 (59.4)	543 (57.0)
High (≥ 3)	2524 (25.5)	668 (39.6)	543 (40.6)	349 (43.0)
BMI^a^				
< 18.5	101 (1.2)	15 (1.1)	9 (0.9)	6 (0.7)
18.5– < 25	2389 (21.5)	297 (17.9)	229 (18.3)	168 (22.0)
25– < 30	4669 (37.4)	604 (31.3)	475 (30.9)	287 (32.5)
≥ 30	5281 (39.9)	1012 (49.7)	831 (49.9)	432 (44.8)
Smoking status^a^				
Never	7640 (61.7)	1068 (57.6)	896 (59.4)	521 (59.5)
Former	2428 (17.0)	381 (16.4)	288 (15.7)	147 (14.6)
Current	2386 (21.3)	486 (26.0)	363 (24.9)	226 (25.9)
Ferritin (μg/L)^a^				
< 12	11,817 (96.8)	1847 (97.2)	1474 (97.1)	854 (97.9)
≥ 12	431 (3.2)	61 (2.8)	49 (2.9)	26 (2.1)
Doctor visits in past 12 months^a^				
0	3566 (32.5)	392 (25.0)	330 (25.4)	254 (31.9)
1	1976 (16.3)	252 (14.3)	216 (15.7)	141 (17.3)
2–3 times	3017 (23.9)	449 (24.1)	371 (24.2)	212 (22.1)
≥ 4	3770 (27.3)	817 (36.6)	613 (34.7)	279 (28.7)
Total number of missing teeth^a^				
0	3577 (40.7)	502 (38.7)	446 (41.9)	338 (49.5)
1–4	4238 (34.3)	585 (34.3)	494 (35.1)	272 (31.8)
5–8	1771 (11.8)	283 (11.3)	215 (10.2)	102 (9.2)
≥ 9	2021 (13.2)	377 (15.7)	271 (12.8)	121 (9.5)
Anti-*H. pylori* antibodies				
Seronegative	4910 (43.4)	915 (48.5)	723 (47.6)	437 (50.6)
Seropositive	7561 (56.6)	1022 (51.5)	825 (52.4)	457 (49.4)
Hepatitis A				
Seronegative	2132 (23.4)	534 (34.8)	441 (35.4)	316 (40.0)
Seropositive	10,339 (76.6)	1403 (65.2)	1107 (64.6)	578 (60.0)

^a^Categories do not sum to the total due to missing data

^b^Percentages are weighted by the sample weights

^c^Self-reported physician diagnosed asthma

^d^Self-reported physician diagnosed asthma without COPD (post-bronchodilator FEV1/FVC ratio  <  0.70)

^e^Childhood onset of asthma (≤ 15 years of age)

### Associations of physician-diagnosed asthma with H. pylori seropositivity

**Fig. 2:**
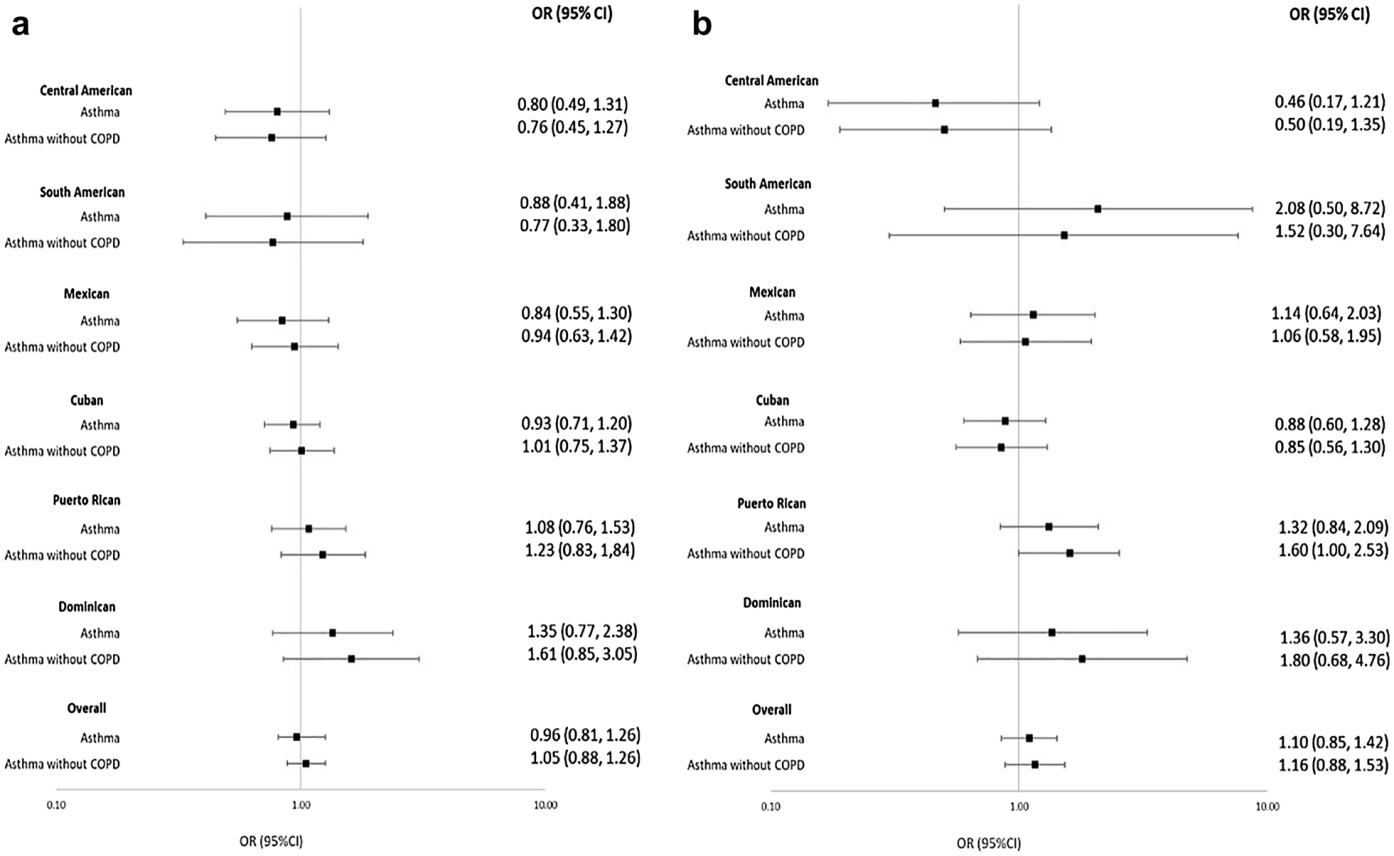
Multivariable-adjusted odds ratios (OR) and 95% confidence intervals (CI) for self-reported physician-diagnosed asthma with *Helicobacter pylori* seropositivity (**a**) and hepatitis A virus seropositivity (**b**). ORs adjusted for age, sex, education, nativity, language acculturation, number of missing teeth, doctor’s visits, ferritin, and body mass index. The overall ORs were also adjusted for Hispanic/Latino background

A multivariable-adjusted logistic regression analysis showed no association between *H. pylori* seropositivity and physician-diagnosed asthma overall and by Hispanic/Latino background (Fig. 2). Furthermore, there was no evidence of effect modification by smoking (p values ranged from 0.2 to 0.9) and nativity (p values  = 0.1–1.0).

### Associations of physician-diagnosed asthma with HAV seropositivity

We found no associations between HAV seropositivity and physician-diagnosed asthma overall and by Hispanic/Latino background in the multivariable-adjusted analysis (Fig. 2). There was no evidence of the effect modification by any of the variables examined (smoking, p values  = 0.3–1.0; nativity, p values  = 0.1–0.8).

Our secondary analyses also found no associations of *H. pylori* and HAV seropositivity with current asthma, childhood onset of asthma, wheezing and hay fever.

## Discussion

Our large study in US Hispanic/Latino populations with variable burdens of asthma and common fecal–oral infections found no association between *H. pylori* or HAV seropositivity with physician-diagnosed asthma, either for all individuals combined or for any of the six specific background groups. Unique features of our study are the consideration of COPD, wheeze and hay fever, and the evaluation of effect modification by smoking and nativity.

Our null results are consistent with two large cross-sectional studies in adults. A study by Fullerton et al. in the United Kingdom (n  = 2437) that reported no association between *H. pylori* seropositivity and several measures of asthma, atopy and lung function [12]. Another study within the Third National Health and Nutrition Examination Survey (n  = 7663) that reported no overall association between *H. pylori* seropositivity and current asthma. However, they found an inverse association for seropositivity to the protein encoded by the cytotoxin-associated gene A, particularly in young adults and early-onset asthma [13].

Asthma is considered a multifactorial disease triggered by a complex environment-host interaction [1], and the specific risk factors might vary across different populations. The hygiene hypothesis links microbial and environmental exposures in early life to the prevalence of asthma, but the mechanisms explaining this association are unclear. Adding to the lack of understanding, growing evidence extends the traditional notion of the hygiene hypothesis to include prenatal exposure[14, 15]. Thus, a potential explanation for our null results may be that *H. pylori* and HAV exposures may not parallel the critical period that would train the immune system. Alternatively, *H. pylori* and HAV infections may be weak surrogates for the hygiene hypothesis.

A recent review of the role of the hygiene hypothesis in asthma concluded that the association remain inconsistent [16]. The authors argue that despite several meta-analyses showing a weak inverse association, individual observational studies that tested the hypothesis in populations with poor hygiene and low *H. pylori* prevalence were unable to confirm a protective association. They concluded that *H. pylori* infection is a marker of poor hygiene rather than an etiologic factor in the development of asthma.

Most Hispanic/Latino backgrounds in HCHS/SOL have high seroprevalences of both *H. pylori* and HAV infections. Despite an existing vaccine for HAV, it is likely that HCHS/SOL participants acquired HAV naturally, as many of the countries from which these populations migrated have intermediate levels of HAV endemicity without national immunization programs.

Our study has several strengths, including a large, diverse, and well-characterized sample of Hispanics/Latinos living in the US. Although HCHS/SOL was not designed to be nationally representative, currently it is the most generalizable cohort of Hispanics/Latinos in the US [17]. Another strength of our study is the robustness of the overall finding; asthma associations without COPD and all subgroup analyses were also null. In addition, our analysis accounted for a wide variety of sociodemographic, clinical and lifestyle factors. Conversely, study limitations include the cross-sectional design that precludes establishing causality and potential misclassification of self-reported disease status. However, a study found that data collected using self-administered questionnaire are a reliable source of information about lifetime and asthma history and age at asthma diagnosis [18]. Also, unfortunately, we were unable to examine other common infections and determinants of poor hygiene in childhood.

## Conclusion

In conclusion, our large serology study did not provide support for the role of *H. pylori* or HAV, as evidence of the hygiene hypothesis in asthma in the diverse Hispanic/Latino populations of the HCHS/SOL study.

## Data Availability

The data that support the findings of this study are available from HCHS/SOL, but restrictions apply to the availability of these data, which were used under license for the current study, and so are not publicly available. Data are however available from the authors upon reasonable request and with permission of HCHS/SOL Steering Committee.

## Abbreviations

HCHS/SOL: Hispanic Community Health Study/Study of Latinos
HAV: Hepatitis A virus
COPD: Chronic obstructive pulmonary disease
